# Melatonin Promotes the Chilling Tolerance of Cucumber Seedlings by Regulating Antioxidant System and Relieving Photoinhibition

**DOI:** 10.3389/fpls.2021.789617

**Published:** 2021-12-09

**Authors:** Xiaowei Zhang, Yiqing Feng, Tongtong Jing, Xutao Liu, Xizhen Ai, Huangai Bi

**Affiliations:** State Key Laboratory of Crop Biology, Key Laboratory of Crop Biology and Genetic Improvement of Horticultural Crops in Huanghuai Region, College of Horticulture Science and Engineering, Shandong Agricultural University, Tai’an, China

**Keywords:** melatonin, reactive oxygen species, antioxidant system, photosynthesis, chilling stress, cucumber

## Abstract

Chilling adversely affects the photosynthesis of thermophilic plants, which further leads to a decline in growth and yield. The role of melatonin (MT) in the stress response of plants has been investigated, while the mechanisms by which MT regulates the chilling tolerance of chilling-sensitive cucumber remain unclear. This study demonstrated that MT positively regulated the chilling tolerance of cucumber seedlings and that 1.0 μmol⋅L^–1^ was the optimum concentration, of which the chilling injury index, electrolyte leakage (EL), and malondialdehyde (MDA) were the lowest, while growth was the highest among all treatments. MT triggered the activity and expression of antioxidant enzymes, which in turn decreased hydrogen peroxide (H_2_O_2_) and superoxide anion (O_2_^⋅–^) accumulation caused by chilling stress. Meanwhile, MT attenuated the chilling-induced decrease, in the net photosynthetic rate (Pn) and promoted photoprotection for both photosystem II (PSII) and photosystem I (PSI), regarding the higher maximum quantum efficiency of PSII (Fv/Fm), actual photochemical efficiency (ΦPS_II_), the content of active P700 (ΔI/I_0_), and photosynthetic electron transport. The proteome analysis and western blot data revealed that MT upregulated the protein levels of PSI reaction center subunits (PsaD, PsaE, PsaF, PsaH, and PsaN), PSII-associated protein PsbA (D1), and ribulose-1,5-bisphosphate carboxylase or oxygenase large subunit (RBCL) and Rubisco activase (RCA). These results suggest that MT enhances the chilling tolerance of cucumber through the activation of antioxidant enzymes and the induction of key PSI-, PSII-related and carbon assimilation genes, which finally alleviates damage to the photosynthetic apparatus and decreases oxidative damage to cucumber seedlings under chilling stress.

## Introduction

For warm climate crops, chilling is considered as a considerable challenge among all abiotic stresses. The previous study showed that low-temperature stress mainly affects light energy utilization and photosynthetic efficiency by disrupting electron transport chains in chloroplasts and mitochondria, leading to reactive oxygen species (ROS) accumulation (Fan et al., 2015). ROS of cells have a strong affinity for membranes, DNA, proteins, carbohydrates, and lipids in plant cells (Anjum et al., 2015; Jajic et al., 2015) and eventually induce cell membrane damage in plants, which severely affects growth and development (Fan et al., 2015; Kazemi-Shahandashti and Maali-Amiri, 2018; Ma et al., 2018). Therefore, it is particularly important to remove excessive ROS in cells. Plants have developed protective mechanisms to regulate the balance between frequent production and scavenging of ROS (Rai et al., 2012). Plants can improve antioxidant enzyme activities to resist the harmful effects of excessive accumulated ROS (Hasanuzzaman et al., 2012). Previous studies have shown that excess ROS induced by chilling stress causes an oxidative damage to plants, which first activates the antioxidant system to respond to oxidative stress. For instance, chilling stress upregulates the gene expression and activities of superoxide dismutase (SOD), catalase (CAT), peroxidase (POD), ascorbate peroxidase (APX), and glutathione reductase (GR) and also the antioxidants such as ascorbate and glutathione (Pan et al., 2020; Zhang et al., 2020). Thus, improving antioxidant capacity is considered as an important method for increasing the chilling tolerance of plants.

Photosynthesis is particularly sensitive to metabolism and developmental processes which can be affected by cold stress (Barrero-Gil et al., 2016). For instance, chilling stress causes the obvious decrease in photosystem II (PSII) and photosystem I (PSI) activity, which inhibits the electron transfer chain and further leads to the generation of singlet oxygen (^1^O_2_) and superoxide in the thylakoid membrane (Terashima et al., 1998; Asada, 2006; Tikkanen et al., 2015; Zhuang et al., 2019). Additionally, the decrease in photosynthetic enzyme activity and messenger RNA (mRNA) abundance, such as ribulose-1,5-bisphosphate carboxylase or oxygenase (Rubisco), transketolase (TK), fructose-1,6-bisphosphate aldolase (FBA), and sedoheptulose-1,7- bisphosphatase (SBPase), are the main reasons for the reduction in photosynthesis under chilling stress (Bi et al., 2015, 2017; Ding et al., 2016; Cai et al., 2018). Thus, how to improve the photosynthetic capacity of chilling sensitive plants under low temperature is an important area of research.

Plants sense chilling signals through hormones to further induce physiological and biochemical reactions (Eremina et al., 2016). Among the different plant hormones, melatonin (*N*-acetyl-5-methoxytryptamine, MT) is a new type of hormone-like substance. It is a small molecule indole that was first discovered in the bovine pineal gland in Lerner et al. (1958). The effects of MT on animals were then studied such as circadian rhythm, mood, sleep and body temperature, physical activity, food intake, retinal physiology, sexual activity, seasonal reproduction, and the immune system (Maronde and Stehle, 2007; Pandi-Perumal et al., 2008; Jan et al., 2009; Hardeland et al., 2012; Carrillo-Vico et al., 2013). The existence of MT in higher plants was demonstrated in 1995 (Dubbels et al., 1995; Hattori et al., 1995). Subsequently, the vital role of MT in plants was gradually reported, such as simulating growth by promoting lateral root formation, plant height, and leaf surface area (Chen et al., 2018; Erdal, 2019) and also improving seed germination (Marta et al., 2016). Moreover, MT was reported as a signaling molecule that participates in the response of plants to abiotic and biotic stresses, which can also stimulate an increase in endogenous MT concentration (Zuo et al., 2014; Shi et al., 2015; Li et al., 2016; Arora and Bhatla, 2017). In 2004, MT was first reported to relieve the chilling stress damage of carrot suspension cells (Lei et al., 2004). Subsequently, MT was found to stimulate the germination of cucumber seeds by protecting the membrane structure against peroxidation under chilling stress (Posmyk et al., 2009). Within cucumber, 200 μmol⋅L^–1^ MT significantly promoted the activities of SOD, APX, GR, monodehydroascorbate reductase (MDHAR), and dehydroascorbate reductase (DHAR), which are involved in the ascorbate-glutathione cycle and accelerated the scavenging of ROS under chilling stress (Zhao et al., 2016). More importantly, MT promoted the chilling tolerance of chlorophyll b-deficient mutant wheat offspring *via* protecting photosynthetic electron transport (Li X. N. et al., 2017).

Cucumber (*Cucumis sativus* L.) is an important vegetable crop that is cultivated worldwide. However, chilling stress severely affects cucumber productivity and quality (Lee et al., 2002; Yu et al., 2002). Previous studies have shown that fruits and even whole seedlings of cucumber produce physiological damage when exposed to chilling stress, especially when they are cultivated in greenhouses during the winter (Cabrera and Saltveit, 1990). Although it has been demonstrated that MT is involved in the antioxidant system and photosynthesis in response to chilling, the optimal concentration and mechanism of regulating the chilling tolerance of cucumber seedlings have not been studied thoroughly. In this study, nutrient solution culture was adopted to explore the effects of different concentrations of MT on cucumber growth and physiological metabolism after chilling stress. We found that MT treatment could improve cold resistance and the effect of 1.0 μmol⋅L^–1^ MT was optimal. For the mechanism of regulating chilling tolerance, proteomics analysis and western blot were used to further study PSI and PSII reaction center activities. The study sheds light on the molecular and physiological mechanism by which MT responds to chilling stress.

## Materials and Methods

Cucumber (Jinyou 35) seeds (bought from Tianjin Kerun Cucumber Research Institute, Tianjin, China) were germinated on petri dishes (8.6 cm in diameter) with moisture filter paper in the dark at 28°C for 1 day, and then germinated seeds were sowed to the nutritional box (8.0 cm in diameter and 8.0 cm in height) filled with base material in a solar greenhouse. The seedlings were routinely managed. The conditions were as follows: maximum of 800–1000 μmol m^–2^⋅s^–1^ photon flux density (PFD) and 25–31°C or 13–21°C day or night temperature under a 13-h photoperiod. At the one-leaf-stage, the seedlings were transferred to black plastic containers (36.5 cm in length, 27.5 cm in width, and 11 cm in height) with 1/2 Hoagland nutrient solution and then 0 μmol⋅L^–1^, 0.3 μmol⋅L^–1^, 0.6 μmol⋅L^–1^, 1.0 μmol⋅L^–1^, 1.5 μmol⋅L^–1^, and 2.0 μmol⋅L^–1^ MT were added until cucumber seedlings were at the two-leaf stage. Twenty-four hours later, all seedlings were exposed to low temperatures (8°C/5°C day or night) and the seedlings treated with 0 μmol⋅L^–1^ MT (H_2_O) were used as the control. Then, leaf samples were taken at 0, 1, 3, and 5 days from 4 to 5 plants (*n* = 3) for the following analyses.

### Measurement of Leaf Area

Leaf area was determined as described previously (Gong and Xiang, 2001).

### Measurement of Malondialdehyde, Electrolyte Leakage, and Chilling Injury Index

Malondialdehyde (MDA) content was measured using the thiobarbituric acid (TBA) colorimetric method (Heath and Packer, 1968). Electrolyte leakage (EL) was estimated as described by Dong et al. (2013). For chilling injury index examination, the seedlings were graded according to the standard described by Semeniuk et al. (1986), and the chilling injury index was calculated using the following equation: chilling injury index = Σ(plants of different grade × grade)/[total plants × 5 (the maximum grade)].

### Determination of Reactive Oxygen Species

Cellular hydrogen peroxide (H_2_O_2_) was clearly visible with an inverted fluorescence microscope using the H_2_O_2_ fluorescent probe 2’, 7’-dichlorodihy drofluorescein diacetate (H_2_DCFDA) (MCE, Cat. No. HY-D0940, Shanghai, China) according to Galluzzi and Kroemer (2014). The leaf disks (0.6 cm in diameter) of the seedlings subjected to the various treatments were soaked in 20 mM HEPES-NaOH buffer (containing 20 μMH_2_O_2_ fluorescent probe, pH 7.5) for 30 min under dark conditions at 25°C. After rinsing with the HEPES-NaOH buffer three times (15 min each time), the H_2_O_2_ production was visual in the form of green coloration (excitation at 488 nm and emission at 522 nm) under an inverted fluorescence microscope (Leica DMi8; Leica, Germany). Cellular superoxide anion (O_2_^⋅–^) was detected using dihydroethidium (DHE) (Fluorescence Biotechnology Co. Ltd, Cat. No. 15200, Beijing, China) as described by Galluzzi and Kroemer (2014). The leaf disks (0.6 cm in diameter) were soaked in 10 mM Tris–HCl buffer (containing 10 μM DHE, pH 7.5) and placed at 37°C in darkness for 30 min. After dying, the samples were washed two times (15 min each time) using Tris–HCl buffer. The orange-red fluorescence of O_2_^⋅–^ (excitation at 490 nm and emission at 520 nm) was clearly visible with the inverted fluorescence microscope.

The quantitation of H_2_O_2_ content was performed according to the instructions specified in the plant H_2_O_2_ Assay Kit (Nanjing Jiancheng Bioengineering Institute, Nanjing, China). The O_2_^⋅–^ production rate was determined using the method presented by Wang and Luo (1990).

### Activity of Antioxidant Enzymes Assay

Cucumber leaves (0.5 g) were ground using 3 ml phosphate buffer (50 mM, pH 7.8) and centrifuged the resulting extract at 12,000 *g* at 4°C for 20 min. The supernatant was reserved at 4°C and prepared to measure the activities of antioxidant enzymes (Cho and Park, 2000). SOD activity was determined according to Beyer and Fridovich’s (1987) method. POD activity was measured with the method of Omran (1980). CAT activity was assayed according to Maehly and Chance (1955). APX activity was detected according to Nakano and Asada (1987), and GR activity was assayed used the method of Foyer and Halliwell (1976).

### Gas-Exchange Parameters Assay

The gas-exchange parameters were assayed with a photosynthetic instrument (Ciras-3, PP-systems International, Hitchin, Hertfordshire, United Kingdom) and controlled PFD (600 μmol⋅m^–2^⋅s^–1^), CO_2_ concentration (360–380 mg⋅L^–1^), and leaf temperature (25°C ± 1°C) from the beginning to end.

### Measurements of Chlorophyll Fluorescence Imaging

Chlorophyll fluorescence imaging of the cucumber seedlings placed in the dark for 45 min was visualized using a chlorophyll fluorescence imaging system (Imaging PAM, Walz, Wurzburg, Germany) with a computer-operated PAM control system (Tian et al., 2017).

### Chlorophyll a Fluorescence Transient and 820 nm Transmission Assay

The Chlorophyll a Fluorescence Transient (OJIP) curve and 820 nm transmission were assayed according to the method of Liu et al. (2020). Leaves were acclimated in the dark for approximately 40 min and measured with an integral multifunctional plant efficiency analyzer (M-PEA, Hansatech, King’s Lynn, Norfolk, United Kingdom). Based on the method of Strasser et al. (2010), the O-J segment of the OJIP curve was standardized. O is the minimum fluorescence, K is 300 μs, and J is 2 ms. Calculation of fluorescence parameters: O-J section standardization: V_*O–J*_ = (F_*t*_-F_0_)/(F_*J*_-F_0_); △V_*O–J*_ = V_*O–J*_-V_*O–J*_ (control); number of reaction centers per unit area *RC/CS_*m*_* = (ABS/CS_*m*_)/(ABS/RC); efficiency of electron moving beyond QA (ψ_0_) = ET/TR = 1-V_*J*_; the capacity of electron transport from PSII to PSI φE_0_ = ET_0_/ABS = (1-F_0_/F_*m*_) × ψ_0_ (Bi et al., 2016). To measure the relative content of the active PSI reaction center, ΔI/I_0_ was measured according to Zhang et al. (2011) and computational formula is as follows (Salvatori et al., 2014): ΔI/I_0_ = (I_0_-I_*m*_)/I_0_ (I_0_, the initial reflection signal between 0.4 and 10 ms; I_*m*_, minimum reflection signal under 820 nm far-red illumination).

### Sodium Dodecyl Sulfate-Polyacrylamide Gel Electrophoresis and Immunoblot Analysis

To extract total protein, 0.2 g leaf samples were ground with liquid nitrogen and extraction buffer (20 mM tricine, 1 mM sodium ascorbate, 400 mM sorbic alcohol, 10 mM NaHCO_3_, 5 mM EDTA⋅Na_2_, and 5 mM MgCl_2_) was added. After centrifugation at 2,000 *g* for 15 min, 5 × loading buffer (CW0027S, Beijing ComWin Biotech Co., Ltd., Beijing, China) was added and then boiled at 100°C for 15 min. 10% sodium dodecyl sulfate-polyacrylamide gel electrophoresis (SDS-PAGE) gel was prepared to separate proteins. Antibodies specific to the PsbA (D1), PSI reaction center subunit II (PsaD), ribulose-1,5-bisphosphate carboxylase or oxygenase (Rubisco) large subunit (RbcL), and Rubisco activase (RCA) proteins (ATCG00020, AT1G03130, ATCG00490, AT2G39730, PhytoAB company, San Francisco, CA, United States) were used to detect D1, PsaD, RbcL, and RCA and followed by incubation with horseradish peroxidase-conjugated anti-rabbit IgG antibody (ComWin Biotech Co., Ltd., Beijing, China). The eECL Western Blot Kit (CW00495, ComWin Biotech Co., Ltd., Beijing, China) was used to detect immune responses, and the ChemiDoc™ XRS imaging system (Bio-Rad Laboratories, Inc., Hercules, CA, United States) was used to record the chemiluminescence.

### Tandem Mass Tag Quantitative Proteomics Analysis

Samples were obtained after 9 h of chilling stress and then fully ground with liquid nitrogen and extracted with lysis buffer (containing 10 mM dithiothreitol and 1% protease inhibitor). An equal volume of Tris-balanced phenol was added and centrifuged at 5,500 *g* at 4°C for 10 min. The supernatant was retained and 5-fold volume of 0.1 M ammonium acetate or methanol was added to precipitate overnight. The precipitates were washed with methanol and acetone, respectively, and redissolved with 8 M urea.

Dithiothreitol was added to the protein solution to make a final concentration of 5 mM and then reduced at 56°C for 30 min. Then, iodoacetamide was added to a final concentration of 11 mM and incubated at room temperature for 15 min in darkness. The urea concentration of the sample was diluted to less than 2 M. Trypsin was added in a 1:50 mass ratio (trypsin: protein), and enzymatic hydrolysis was performed overnight at 37°C. Trypsin was added at a mass ratio of 1:100 (trypsin: protein) again, and enzymatic hydrolysis was continued for 4 h. After trypsin digestion, the peptides were desalted with a Strata X C18 SPE column (Phenomenex) and then vacuum freeze-dried. The peptides were dissolved in 0.5 M triethylammonium bicarbonate (TEAB) and labeled according to the instructions of the Tandem Mass Tag (TMT) kit. The peptides were separated with high pH reverse-phase high-performance liquid chromatography (HPLC) using a Thermo Betasil C18 column (5 μm particles, 10 mm ID, 250 mm length), dissolved with mobile phases A (0.1% formic acid), and separated by an EASY-nLC 1000 UPLC system. Mobile phase A was aqueous solution containing 0.1% formic acid, and mobile phase B was acetonitrile solution containing 0.1% formic acid. Liquid phase gradient setting was as follows: 0–43 min, 6–22% phase B; 43–56 min, 22–30% phase B; 56–58 min, 30–80% phase B; 58–60 min, 80% phase B, and the flow rate maintained at 300 nL/min. The peptides were separated by a UPLC system and then injected into a capillary ion source for ionization and analyzed by times TOF Pro mass spectrometry. The ion source voltage was set at 1.4 kV, and the peptide parent ions and their secondary fragments were detected and analyzed using TOF. The scanning range of secondary mass spectrometry was set to 100–1700 m/z. The data acquisition mode was parallel accumulation serial fragmentation (PASEF) mode. Secondary mass spectrometry data were retrieved using the Maxquant search engine (v1.6.5.0), and peptide length was analyzed with mass spectrometry. Finally, bioinformatics analysis was performed. The quantitative proteins were identified and analyzed with threshold value of differential expression change of 1.2-fold and a statistically tested *t*-test *p*-value < 0.05.

### RNA Extraction and Gene Expression Analysis

Cucumber leaves were ground thoroughly with liquid nitrogen, and total RNA was extracted with TransZol reagent (Transgen, Beijing, China). First, 0.2 g ground powder was added 1 ml TransZol and then mixed. After standing for 5 min, 0.2 ml chloroform was added followed by shaking vigorously for 15 s and then standing for 3 min at room temperature. The solution was centrifuged at 10,000 *g* and 4°C for 15 min, and the supernatant was retained. Then, 0.5 ml isopropyl alcohol was added to the supernatant, mixed gently, and incubated for 10 min at room temperature. The sample was centrifuged at 10,000 *g* and 4°C for 10 min to remove the supernatant, and then 1 ml of 75% ethanol was added to the precipitate. Oscillation and centrifugation were performed at 7,500 *g* and 4°C for 5 min. The supernatant was removed, and the precipitation was dried at room temperature. The precipitate was dissolved in 100 μL RNA solution. The resulting total RNA was reverse transcribed according to the instructions of HiScript^®^ IIIRT SuperMix for qPCR (+gDNA wiper) (Vazyme, Nanjing, China). Genomic DNA was removed using 4 × gDNA wiper Mix at 42°C for 2 min. Then, 5 × HiScript III qRT SuperMix containing all the components required for reverse transcription was mixed with RNA and could be used immediately for PCR at 37°C for 15 min and then 85°C for 5 s. Relative gene expressions were analyzed by real-time quantitative PCR (RT-qPCR) using ChamQ™ Universal SYBR^®^ qPCR MasterMix (Vazyme, Nanjing, China) according to the instructions. The predenaturation was performed at 95°C for 30 s. Then, 40 cycles were performed at 95°C for 10 s and 60°C for 30 s. Finally, 95°C for 15 s and 60°C for 60 s and 95°C for 15 s acquired fusion curves. The cucumber β-actin gene (Gene ID: Solyc11g005330) was used as an internal reference gene. The primers were designed and synthesized by BGItech and were shown in Table 1. qRT-PCR was performed with three biological replicates and three technical replicates.

**TABLE 1:** Primers used in quantitative real-time PCR (qRT-PCR).

Genes	Accession numbers	Primer pairs (5′-3′)
*ACTIN*	DQ115883	CCACGAAACTACTTACAACTCCATC
		GGGCTGTGATTTCCTTGCTC
*SOD*	NM_001280768	GGAAAGATGTGAAGGCTGTGG
		GCACCATGTTGTTTTCCAGCAG
*POD*	XM_004151830	GGTTTCTATGCCAAAAGCTGCCC
		CAGCTTGGTTGTTTGAGGTGGAG
*CAT*	NM_001308916	AATGGCCGGAGGATGTGA
		CCAACGACATAGAGAAAGCCAAC
*APX*	NM_001280706	GTGCTACCCTGTTGTGAGTG
		AACAGCGATGTCAAGGCCAT
*GR*	NM_001308836	TGATGAGGCTTTGAGTTTAGAGGAG
		AACTTTGGCACCCATACCATTC
*PsaD*	Csa_3G147780	TATGTCATAACATGGGAATCC ACCTCAATAGGGCTAACATTCT
		ACCACAACTGGGTATCGAGTGTT
*PsaE*	Csa_2G079660	ATAGAGAAAAAAGATCATTCA
*PsaF*	Csa_1G714680	ATCAAAGCCACGATCGAAAAGAC
		TCACCATTGACAAGCTCTCTG
*PsaH*	Csa_3G483830	AGGCTGGTGCTGTGGTTGCTAAG
		CCACGTGGCCCAAGTTTCGGTGG
*PsaN*	Csa_6G483300	ACTCCTCTGCTAATGCTGGAGT
		TCCTTCCCTTCACACTCCAATT

### Statistical Analysis

The experimental design was a completely randomized block design. The data are presented as the mean ± standard deviation (SD) of three to five replicates. Analysis of variance was conducted using DPS software. Duncan’s multiple range test (DMRT) was applied to statistical analysis among treatments, and the standard for significant difference was *p* < 0.05.

## Results

### Exogenous Melatonin Positively Regulates the Chilling Tolerance of Cucumber Seedlings

**FIGURE 1:**
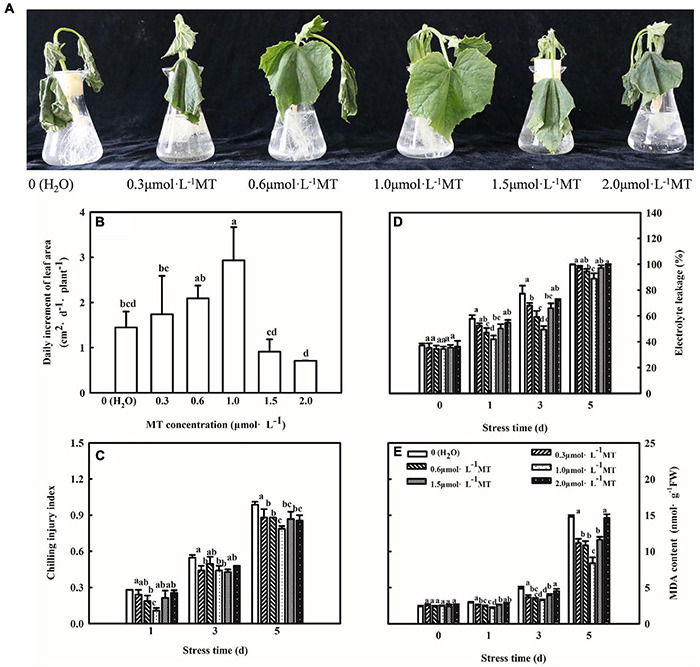
Effect of MT on the chilling tolerance of cucumber seedlings. **(A)** Phenotype of seedlings. **(B)** Daily increment of leaf area. **(C)** Chilling injury index. **(D)** Electrolyte leakage. **(E)** MDA content. The two-leaf stage cucumber seedlings were treated with 0, 0.3, 0.6, 1.0, 1.5, or 2.0 μmol⋅L^–1^ MT, respectively, for 24 h, and then seedlings were exposed to 8 or 5°C for 5 days. All values shown are mean ± SD (*n* = 3). a, b, c, and d indicate that mean values are significantly different among samples (*p* < 0.05).

To study the concentration effect of MT on regulating chilling tolerance of cucumber seedlings, we treated seedlings with 0.3, 0.6, 1.0, 1.5, and 2.0 μmol⋅L^–1^ MT, respectively. As shown in Figure 1A, cucumber seedlings treated with H_2_O showed leaf-wilting and spots at advanced stages of the chilling stress. Furthermore, we examined the change in growth and lipid peroxidation of cucumber seedlings under low-temperature intensity. An increase in the chilling injury index, EL, and MDA content in cucumber seedlings treated with H_2_O was observed along with an increase in stress duration (Figures 1B–E). MT obviously alleviated the chilling injury of cucumber seedlings, as evidenced by the slightly wilted leaf, lower chilling index, EL, MDA content, and higher leaf area. Moreover, the alleviation effect firstly increased and then decreased with increasing concentrations of MT and 1.0 μmol⋅L^–1^ MT showed the best effect. These results illustrated that MT could improve the chilling tolerance of cucumber seedlings in a concentration-dependent manner, and 1.0 μmol⋅L^–1^ MT was used in further experiments.

### Melatonin Stimulates Antioxidative Pathways and Reduces Oxidative Stress

**FIGURE 2:**
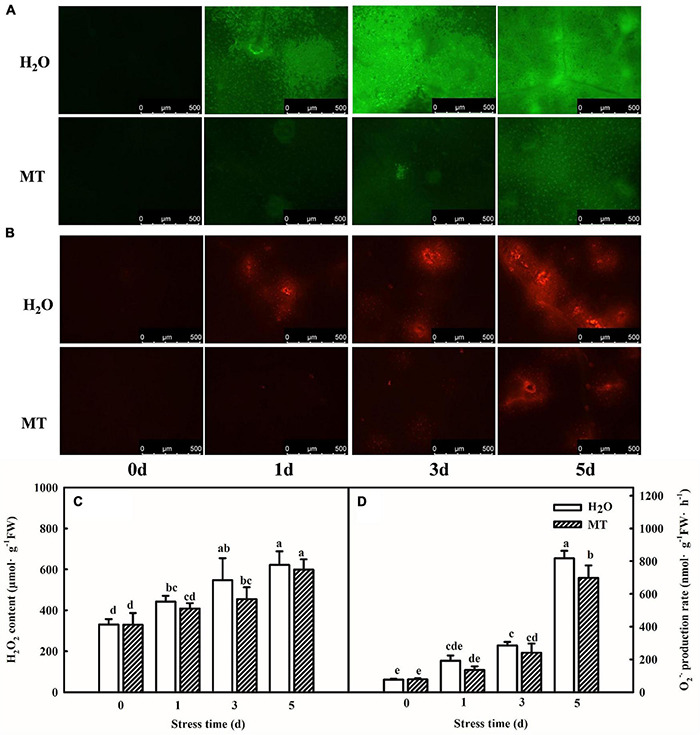
Effect of MT on the accumulation of ROS under chilling stress. **(A)** H_2_O_2_ inverted fluorescence microscope imaging. **(B)** O_2_^⋅–^ inverted fluorescence microscope imaging. **(C)** H_2_O_2_ content. **(D)** O_2_^⋅–^ production rate. The two-leaf stage cucumber seedlings were treated with H_2_O and 1.0 μmol⋅L^–1^ MT, respectively, for 24 h, and then seedlings were exposed to 8 or 5°C for 0–5 days. All values shown are mean ± SD (*n* = 3). a, b, c, d, and e indicate that mean values are significantly different among samples (*p* < *0.05*).

Chilling stress can break the balance of reactive oxygen metabolism, which further results an oxidative damage to plants. Thus, we assessed the effect of exogenous MT on the ROS contents and antioxidant system following chilling stress at 8 or 5°C for 5 days. Importantly, MT accumulated less H_2_O_2_ and O_2_^⋅–^, and inverted fluorescence microscope observations of H_2_O_2_ and O_2_^⋅–^ were consistent with the quantitative determination. For instance, the H_2_O_2_ content of H_2_O-treated seedlings increased by 65.5% but MT-treated seedlings increased by 37.8%, which was obviously lower as compared to H_2_O treatment especially at 3 days of chilling stress (Figure 2).

**FIGURE 3:**
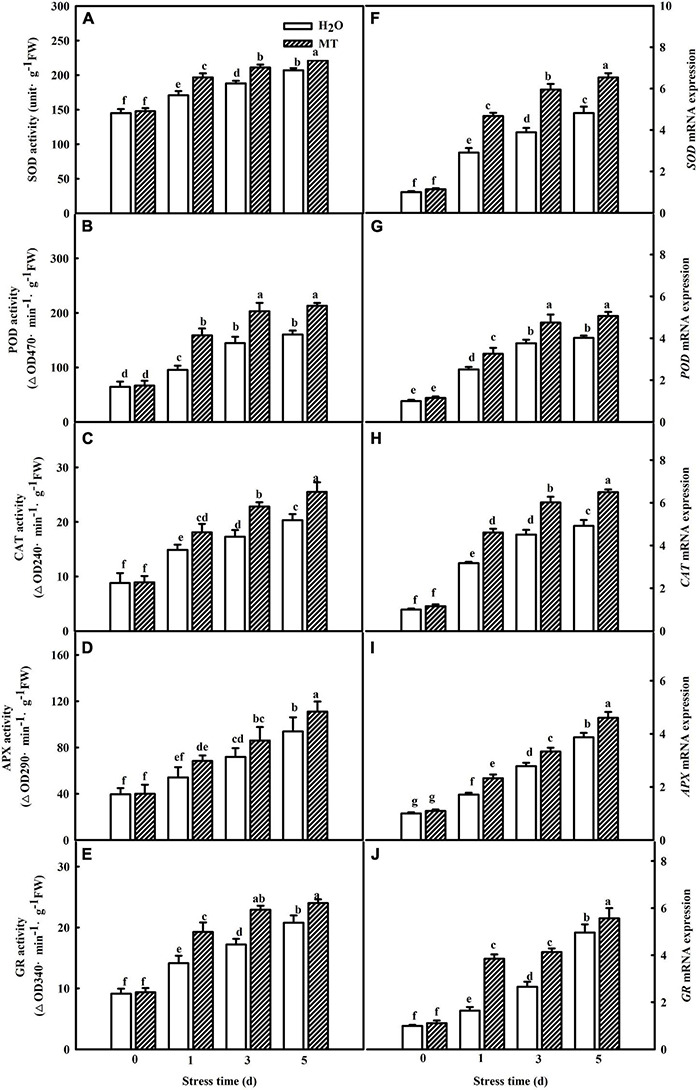
Effect of MT on the activities and relative mRNA expressions of antioxidant enzymes in cucumber seedlings under chilling stress. **(A–E)** Activities of SOD, POD, CAT, APX, and GR, the second leaf was sampled for the activities assay. **(F–J)** Relative mRNA expressions of *SOD*, *POD*, *CAT*, *APX*, and *GR*, total RNA was separately isolated from the same tissues for the determination of activities and subjected to RT-PCR. The two-leaf stage seedlings were treated with H_2_O and 1.0 μmol⋅L^–1^ MT, respectively, for 24 h. Then, the seedlings were exposed to 8 or 5°C for 5 days. All values shown are mean ± SD (*n* = 3). a, b, c, d, e, f, and g indicate that mean values are significantly different among samples (*p* < 0.05).

Moreover, we examined the impacts of MT on the activities and gene mRNA abundance of antioxidant enzymes during chilling stress. As shown in Figures 3A–E, increases in SOD, POD, CAT, APX, and GR activities in both H_2_O- and MT-treated cucumber seedlings were observed with the increasing of chilling stress. Notably, seedlings of MT treatment showed higher activities of the five antioxidant enzymes than the H_2_O treatment during chilling stress. Additionally, the regulation of antioxidant enzyme genes by MT was detected during chilling stress. The mRNA expression levels of *SOD, POD, CAT, APX*, and *GR* were significantly upregulated when the seedlings were exposed to 8 or 5°C for 5 days, which was positively correlated with the increased antioxidant enzyme activities of the chilling-treated seedlings (Figures 3F–J). Consistently, MT-treated seedlings displayed higher mRNA abundance of *SOD*, *POD*, *CAT*, *APX*, and *GR* than H_2_O-treated seedlings, indicating that MT could enhance the antioxidant capacity to protect the membrane against oxidative damage caused by chilling stress.

### Melatonin-Induced Chilling Tolerance Is Associated With Improved Photosynthesis in Cucumber Seedlings

**FIGURE 4:**
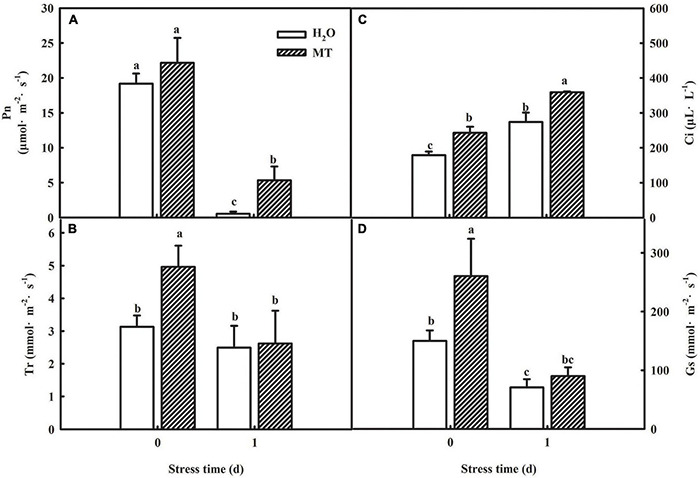
Effect of MT on the gas-exchange parameters of cucumber seedlings under chilling stress. **(A)** Pn; **(B)** Tr; **(C)** Ci; **(D)** Gs. The two-leaf stage cucumber seedlings were treated with H_2_O and 1.0 μmol⋅L^–1^ MT, respectively, for 24 h, and then seedlings were exposed to 8 or 5°C for 1 days. All values shown are mean ± SD (*n* = 3). a, b, and c indicate that mean values are significantly different among samples (*p <* 0.05).

The generation of oxidative stress is closely related to photosynthetic capacity, so we studied the effect of MT on photosynthesis. To determine whether MT could improve the photosynthetic capacity, we examined the changes in gas-exchange parameters in cucumber seedlings treated with H_2_O and MT, respectively. As shown in Figure 4, net photosynthetic rate (Pn) and stomatal conductance (Gs) of cucumber seedlings were significantly reduced, whereas the intercellular CO_2_ concentration (Ci) increased under chilling stress. Additionally, we found that MT significantly relieved the decrease in Pn and Gs induced by chilling stress.

**FIGURE 5:**
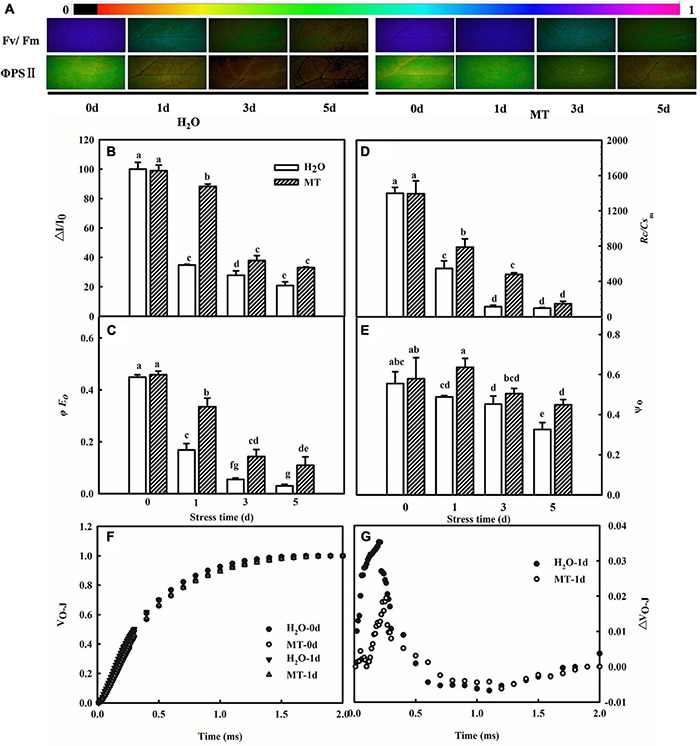
Effect of MT on the activity of PSI and PSII under chilling stress. **(A)** Fv/Fm and ΦPSII; **(B)** △I/I_0_; **(C)** φE_0_; **(D)** *Rc/C*sm; **(E)** Ψ_0_; **(F)** V_*O–J*_; **(G)** △V_*O–J*_. The two-leaf stage cucumber seedlings were treated with H_2_O and 1.0 μmol⋅L^–1^ MT, respectively, for 24 h, and then seedlings were exposed to 8°C or 5°C for 5 days. All values shown are mean ± SD (*n* = 3). a, b, c, d, e, f, and g indicate that mean values are significantly different among samples (*p* < 0.05).

Considering the decrease of Gs accompanied by the increase of Ci, which suggested that the decline of Pn was mainly caused by non-stomatal factors under chilling intensity, we also determined the activities of PSII and PSI in both H_2_O- and MT-treated seedlings. Under normal conditions, no significant differences were observed in higher maximum quantum efficiency of PSII (Fv/Fm) and actual photochemical efficiency between H_2_O and MT treatments. However, when exposed to chilling at 8°C or 5°C for 1 and 3 days, H_2_O and MT treatments showed dramatic decreases in F_*v*_/F_*m*_ and ΦPSII in cucumber seedlings, but the decrease in MT was notably lower than that of H_2_O treatment (Figure 5A). The results showed that chilling stress caused obvious photoinhibition to cucumber seedlings, and exogenous MT could significantly reduce the damage of photoinhibition to cucumber seedlings. Meanwhile, we found that the levels of *Rc/Cs*_*m*,_ Ψ_0_ and φE_0_ decreased in cucumber seedlings following 5 days of chilling stress and that the changes in MT-treated seedlings were much lower (Figures 5C–E). For instance, φE_0_ was suppressed by 62.58% in H_2_O-treated seedlings but only by 26.86% in MT-treated seedlings after 1 days of chilling stress compared to seedlings under normal conditions. The OJIP curve at 300 μs is called the K-point, and the increase in the K-point shows that the oxygen evolution complex (OEC) is damaged under stress. The K-point is a specific marker of photoinhibition of the PSII donor side. To observe the K-point, the O-J segments of the OJIP curves before and after chilling stress were standardized (Figures 5F,G). The results showed that before chilling stress, the curves of the O-J segment of the H_2_O and MT treatments were similar. Following of 1 day of chilling stress, the K-point of cucumber seedlings increased significantly, but the K-point of the MT treatment was significantly lower than that of the H_2_O treatment. Thus, the results showed that exogenous MT alleviated the damage to PSII and increased the efficiency of electron transfer under chilling stress.

Furthermore, PSI was sensitive to abiotic stresses. As shown in Figure 5B, chilling stress led to a decrease in △I/I_0_. Compared to the H_2_O treatment, MT showed markedly higher △I/I_0_ than the H_2_O treatment when seedlings were exposed to 8°C or 5°C for 5 days, demonstrating that MT could upregulate the activity of the PSI reaction center under chilling stress.

### Analysis of Quantitative Proteomic in Cucumber Seedlings in Response to Melatonin Under Chilling Stress

**FIGURE 6:**
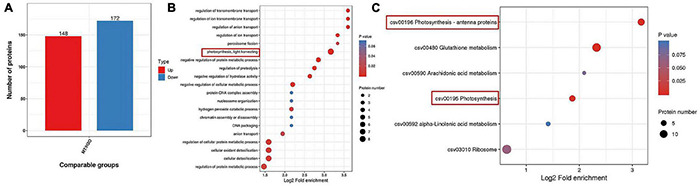
Protein expression comparison **(A)**, Go, and KEGG analysis of differential expressed proteins **(B,C)** in MT vs. H_2_O (*p* < 0.05 and a fold change of >1.2 or <1.2). The two-leaf stage cucumber seedlings were treated with H_2_O and 1.0 μmol⋅L^–1^ MT, respectively, for 24 h, and then seedlings were exposed to 5°C for 9 h. The bubble size represents the number of proteins, and the bubble color means the *p*-value of the significance.

To further confirm the possible mechanism associated with the mitigation of photosynthesis under chilling stress by MT, we detected the protein expression in cucumber seedlings sprayed with H_2_O and MT after 9 h of low-temperature treatment. Based on the criteria for significantly different expressions of *p* < 0.05 and a fold change of >1.2 or <1.2 for proteins in three biological replicates, we identified 320 significantly expressed proteins in MT-treated seedlings as compared to H_2_O-treated seedlings, including 148 upregulated proteins and 172 downregulated proteins compared with H_2_O-treated seedlings under chilling stress (Figure 6A). Furthermore, we mainly focused on the analysis of the 148 upregulated proteins and found that the enrichment degree of upregulated proteins mainly focused on photosynthesis pathways (Figures 6B,C), which were mainly related to PSI and light energy capture.

### Transcript Levels Analysis of Photosystem I-Associated Genes Induced by Melatonin Under Chilling Stress

**FIGURE 7:**
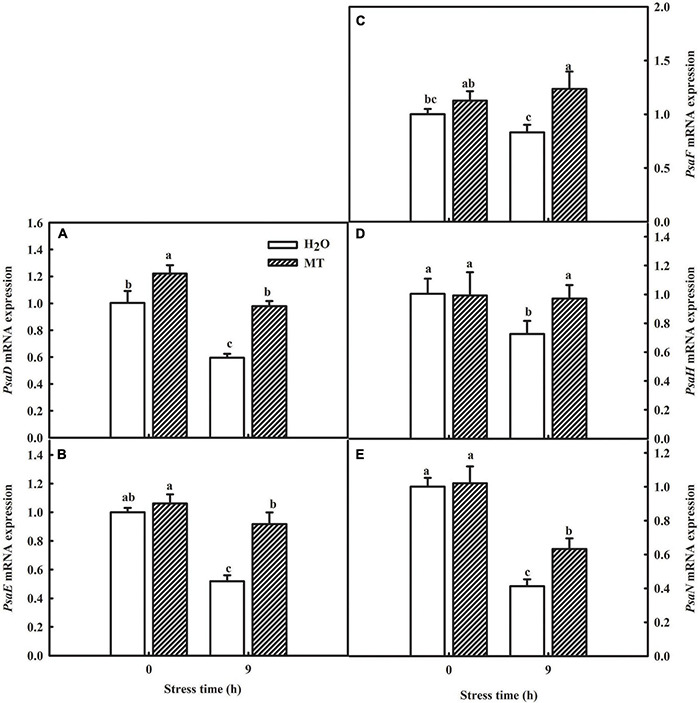
Verification of proteomic results by qRT-PCR. **(A)** mRNA expression of *PsaD*; **(B)** mRNA expression of *PsaE*; **(C)** mRNA expression of *PsaF*; **(D)** mRNA expression of *PsaH*; **(E)** mRNA expression of *PsaN*. The two-leaf stage cucumber seedlings were treated with H_2_O and 1.0 μmol⋅L^–1^ MT, respectively, for 24 h, and then seedlings were exposed to 5°C for 9 h. All values shown are mean ± SD (*n* = 3). a, b, and c indicate that mean values are significantly different among samples (*p* < 0.05).

Table 2 showed a partial list of the differentially expressed proteins related to PSI, such as PsaD, PsaE, PsaF, PsaH, and PsaN. To further validate our quantitative proteomic results, we measured the RNA abundance of *PsaD*, *PsaE*, *PsaF*, *PsaH*, and *PsaN* in cucumber seedlings with quantitative RT-PCR assays after 9 h chilling stress, and Figure 7 displayed significantly higher mRNA abundance of *PsaD*, *PsaE*, *PsaF*, *PsaH*, and *PsaN* in MT-treated seedlings, compared to H_2_O-treated seedlings. The results were highly consistent with the proteomic data, suggesting the confidence of the quantitative proteomic data.

**TABLE 2:** Functional classifications of identified proteins significantly expressed in cucumber seedlings of MT versus H_2_O plants (*p* < 0.05 and a fold change of >1.2 or <1.2).

Protein accession	Protein description	MT/H_2_O Ratio	*P*-value	Gene name	KEGG Gene	Plant species
A0A0A0L4Y6	Photosystem I reaction center subunit II	1.701	1.4495E-06	Csa_3G147780	psaD	Cucumis sativus
A0A0A0LJQ1	Photosystem I reaction center subunit IV	1.732	2.8441E-06	Csa_2G079660	psaE	Cucumis sativus
A0A0A0M3 × 2	Photosystem I reaction center subunit III	1.585	0.000038159	Csa_1G714680	psaF	Cucumis sativus
A0A0A0LAW4	Photosystem I reaction center subunit VI	1.283	0.00136104	Csa_3G483830	psaH	Cucumis sativus
A0A0A0KFM1	Photosystem I reaction center subunit N	1.469	0.000036481	Csa_6G483300	psaN	Cucumis sativus

### Western Blot Analysis of Photosynthesis-Associated Proteins Induced by Melatonin Under Chilling Stress

**FIGURE 8:**
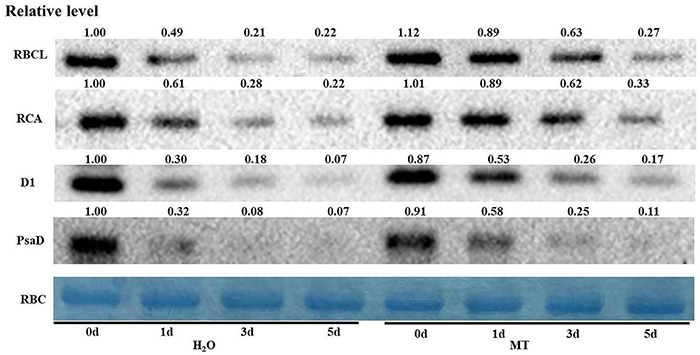
Effect of MT on the protein expression of RBCL, RCA, D1, and PsaD of cucumber seedlings under chilling stress. The two-leaf stage cucumber seedlings were treated with H_2_O and 1.0 μmol⋅L^–1^ MT, respectively, for 24 h, and then seedlings were exposed to 8 or 5°C for 5 days. The leaves for photosynthesis analysis were sampled for total protein extraction. The Rubisco (RBC) was used as an internal reference protein to adjust the concentration of different samples, and the value of H_2_O treatment at 0 days was set to 1.

Key enzymes in the reaction stage and PSII-related enzymes are important factors which that affect photosynthesis. Thus, we chose certain key proteins involved in the photosynthetic system for western blot, and the data showed that the protein levels of RBCL, RCA, and D1 in H_2_O-treated seedlings decreased significantly with increasing chilling stress time (Figure 8); however, MT obviously relieved the degradation of RBCL, RCA, and D1 induced by chilling stress. The protein level of PsaD was consistent with the proteomic data (Figure 8). The results further suggested the regulatory mechanism of MT on photosynthesis under chilling stress.

## Discussion

Previous studies have revealed that MT was involved in tolerance to multiple abiotic stresses, including tolerance to chilling, drought, salt, and cadmium stress (Bajwa et al., 2014; Zuo et al., 2014; Li et al., 2016; Marta et al., 2016; Arora and Bhatla, 2017; Aghdam et al., 2020). In this study, the cucumber seedlings pretreated with different concentrations of MT showed changes of varying degrees in chilling injury index, EL, and MDA under chilling stress, especially with 1.0 μmol⋅L^–1^ MT treatment (Figure 1). These findings confirmed that MT could enhance the chilling tolerance of cucumber seedlings in a concentration-dependent manner. More importantly, MT easily showed light decomposition, and here, we added MT to the liquid nutrient solutions but not foliar application, which was better avoiding the light degradation of MT and decreasing the effective application amount of MT. For instance, MT can promote the chilling tolerance of cucumber seedlings at 100 μmol⋅L^–1^ when MT is applied as a spray (Feng et al., 2021).

Ninety-five percent of the dry matter in plants comes from the photosynthesis, which is the basis for the yield. Different lines of evidence highlight the effect of MT on photosynthesis of plants (Li H. et al., 2017; Liang et al., 2019; Yan et al., 2021). MT could trigger many genes and enzymes associated with carbon and nitrogen metabolism which in turn improved photosynthesis to promote plant growth and development under abiotic stress (Debnath et al., 2019; Iqbal et al., 2021; Ren et al., 2021). Here, we found that cucumber seedlings in the MT treatment showed a higher daily increase in leaf area than those in the H_2_O treatment under chilling stress, which was related to the higher Pn of the MT treatment following 1 day of chilling stress (Figure 4A). However, evidence showing the regulatory mechanisms of MT on photosynthesis in plant tolerance to low-temperature intensity, particularly in chilling-sensitive crop species, is limited. Previous studies showed that the decline in Pn during chilling stress was mainly due to the decrease of activity in photosynthetic enzyme and also PSII and PSI (Bi et al., 2015; Zhang et al., 2016). Under chilling stress, PSII activity, as one of the most notable hallmarks, obviously decreases, which has been proved to contain at least 20 different subunits, and D1 protein of PSII is the most attack site under various abiotic stresses (Kong et al., 2014; Gao et al., 2018; Zhuang et al., 2019). Similarly, we found that the low-temperature intensity led to a decrease in Fv/Fm and ΦPSII (Figure 5A); however, the application of MT in cucumber seedlings maintained the higher PSII activity than that in H_2_O-treated seedlings at the end of the stress (Figure 5A). The study of the reaction center of PSII and its donor and acceptor sides are considered as important for PSII activity of plants, especially under abiotic stress (Krause and Weis, 1991; Maxwell and Johnson, 2000; Li et al., 2005). In this paper, we observed that MT obviously alleviated the decline of *Rc/Cs_*m*_*, Ψ_0_, φE_0_ and increase of △V_*O–J*_ (Figures 5C–G), implying MT increased the amount of the reaction center, decreased chilling injury to the OEC, and finally protected the electron transfer during chilling stress. PSI in chilling-sensitive plants was thought to be more sensitive than PSII under chilling stress (Terashima et al., 1994; Zhang et al., 2011), and the decrease in PSI was mainly due to the decline in enzyme activity in the dark reaction during the photosynthetic process, further resulting in an increase in the excess excitation energy of PSII under chilling stress (Zhang et al., 2009). In this study, we found that PSI activity was decreased in both MT- and H_2_O-treated seedlings (Figure 5B); however, PSI activity in H_2_O-treated seedlings decreased the most following 5 days of chilling stress, which may be related to the upregulation of RCA and ribulose-1,5-bisphosphate carboxylase or oxygenase (Rubisco) larger subunit protein level by MT (Figure 8). To further provide evidence for the mechanisms by which MT affected photosynthesis to improve the chilling tolerance of cucumber seedlings, we detected the change in proteomic level in cucumber seedlings pretreated with H_2_O and MT under chilling stress. The data showed that MT significantly upregulated 148 proteins that were mainly enriched in photosynthesis pathways following 9 h of chilling stress (Figure 6). Surprisingly, we found that the change of proteins in photosynthesis was mainly related to PSI, such as PsaD, PsaE, PsaF, PsaH, and PsaN (Table 2); however, none PSII-related proteins were found following 9 h of chilling stress. In addition, we found that similar to the change of PSII and PSI activity, the western blot results showed MT alleviated the decline in PsaD and D1 protein following 1 day of chilling stress (Figure 8). Our results demonstrated that MT could upregulate the PSI-related protein level earlier than PSII-related protein during the chilling stress to response to PSI photoinhibition occurred earlier than PSII photoinhibition in chilling-sensitive plants (Terashima et al., 1994).

Reactive oxygen species of plants is homeostasis under normal conditions and excess ROS content induced by abiotic stress resulted in the oxidative damage to plants (Xu et al., 2019). The reports of Mittler et al. (2011) indicated that ROS in plants occurred mainly through 10 pathways, which included photosynthesis, RBOH oxidase, photorespiration, etc. It is generally recognized that photoinhibition induced by abiotic stress is the main reason for ROS accumulation in chloroplast, which further affects the photosynthetic apparatus (Savitch et al., 1997). The decrease of electron transfer at the reduction side of PSI during photosynthetic process is the main reason for ROS accumulation in chloroplast under chilling stress (Sonoike, 1996; Zhang et al., 2014). Here, the lower ROS content in MT-treated seedlings was well associated with an increase in △I/I_0_ in MT-treated seedlings under chilling stress (Figure 5B). Furthermore, it is obvious that plants showed enhanced chilling tolerance are related to the elevating ROS scavenging capability (Luo et al., 2015; Pan et al., 2020; Zhang et al., 2020). In this study, we found that MT markedly enhanced the activities and relative mRNA expression of antioxidant enzymes, including SOD, POD, CAT, APX, and GR in cucumber seedlings (Figure 3), implying MT participated in the modulation of ROS accumulation through upregulating the mRNA abundance of antioxidant defense genes and further promoted the chilling tolerance, which was consistent with the results of previous studies (Hu et al., 2016; Marta et al., 2016).

## Conclusion

**FIGURE 9:**
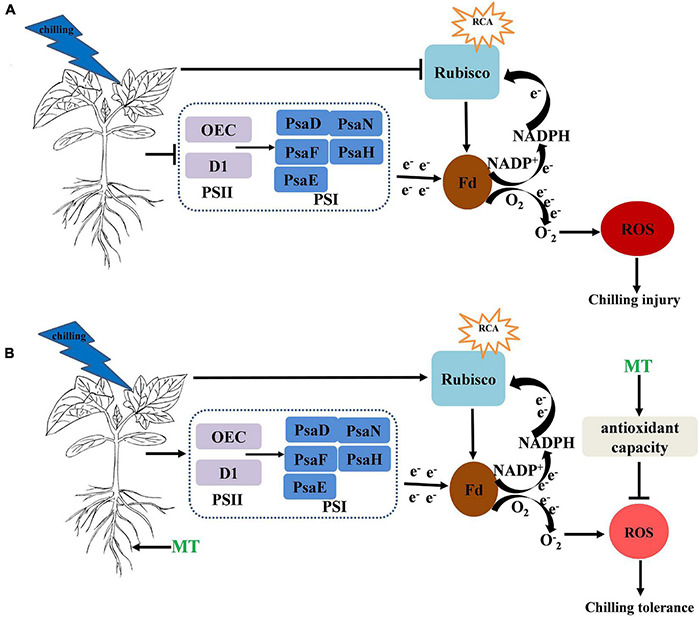
**(A,B)** Simplified schematic model for MT on the regulation of the chilling tolerance in cucumber → indicates upregulation or positive effects. ⊥ Indicates downregulation or negative effects. The circle and color of ROS indicate the change of ROS content under chilling stress and the darker the color and larger the circle, the higher the content of ROS.

In summary, MT induced chilling tolerance in cucumber seedlings, as shown by the decrease in stress-induced electrolyte leakage, the decreased contents of H_2_O_2_, MDA and the production rate of O_2_^⋅–^, which occurred partially due to the induction of antioxidant metabolism. Additionally, the MT treatment maintained a high photosynthetic carbon assimilation capacity, increased the PSII- and PSI-related protein levels, which increased the activity of the PSII and PSI reaction centers and electron transfer efficiency, thus finally alleviated the damage to photosynthetic apparatus under chilling stress (Figure 9).

## Data Availability Statement

The mass spectrometry proteomics data have been deposited to the ProteomeXchange Consortium via the PRIDE (Website: http://www.ebi.ac.uk/pride) partner repository with the dataset identifier PXD029134.

## Ethics Statement

The authors declare that the experiments were performed in compliance with the current laws of China.

## Author Contributions

XZ performed most of the experiments, analyzed the data, and completed the first draft. HB designed the research and edited the study. YF, TJ, XL, and XA worked together with XZ to accomplish the experiment. All authors contributed to the article and approved the submitted version.

## Conflict of Interest

The authors declare that the research was conducted in the absence of any commercial or financial relationships that could be construed as a potential conflict of interest.

## Publisher’s Note

All claims expressed in this article are solely those of the authors and do not necessarily represent those of their affiliated organizations, or those of the publisher, the editors and the reviewers. Any product that may be evaluated in this article, or claim that may be made by its manufacturer, is not guaranteed or endorsed by the publisher.
